# Healthcare institutions’ recommendation regarding the use of FFP-2 masks and SARS-CoV-2 seropositivity among healthcare workers: a multicenter longitudinal cohort study

**DOI:** 10.1186/s13756-021-01047-x

**Published:** 2022-01-10

**Authors:** Katarzyna Szajek, Felix Fleisch, Sandra Hutter, Martin Risch, Theresa Bechmann, Valerie A. Luyckx, Sabine Güsewell, Cédric Hirzel, Alexia Cusini, AMICO Study Group, AMICO Study Group, Vedat Eronat, Luwina Eichweber, Theodor von Fellenberg, Mauro Albertini, Gian Flury, Marie-Charlotte Brüggen, Andres Schneeberger, Thomas Koch, Michele Genoni, Walter Kistler, Patrick Egger

**Affiliations:** 1 Department of Internal Medicine, Cantonal Hospital of Grisons, Chur, Switzerland; 2 Division of Infectious Diseases, Cantonal Hospital of Grisons, 7000 Chur, Switzerland; 3 Central Laboratory, Cantonal Hospital of Grisons, Chur, Switzerland; 4 Division for Information and Communication Technology, Cantonal Hospital of Grisons, Chur, Switzerland; 5 Division of Nephrology, Cantonal Hospital of Grisons, Chur, Switzerland; 6 Clinical Trials Unit, Cantonal Hospital of St. Gallen, St. Gallen, Switzerland; 7 Department of Infectious Diseases and Hospital Epidemiology, University Hospital and University of Bern, Bern, Switzerland

**Keywords:** SARS-CoV-2, Healthcare workers, Seroconversion, FFP-2 and surgical masks, Mask policy

## Abstract

**Background:**

Health care workers (HCW) are heavily exposed to SARS-CoV-2 from the beginning of the pandemic. We aimed to analyze risk factors for SARS-CoV-2 seroconversion among HCW with a special emphasis on the respective healthcare institutions’ recommendation regarding the use of FFP-2 masks.

**Methods:**

We recruited HCW from 13 health care institutions (HCI) with different mask policies (type IIR surgical face masks vs. FFP-2 masks) in Southeastern Switzerland (canton of Grisons). Sera of participants were analyzed for the presence of SARS-CoV-2 antibodies 6 months apart, after the first and during the second pandemic wave using an electro-chemiluminescence immunoassay (ECLIA, Roche Diagnostics). We captured risk factors for SARS-CoV-2 infection by using an online questionnaire at both time points. The effects of individual COVID-19 exposure, regional incidence and FFP-2 mask policy on the probability of seroconversion were evaluated with univariable and multivariable logistic regression.

**Results:**

SARS-CoV-2 antibodies were detected in 99 of 2794 (3.5%) HCW at baseline and in 376 of 2315 (16.2%) participants 6 months later. In multivariable analyses the strongest association for seroconversion was exposure to a household member with known COVID-19 (aOR: 19.82, 95% CI 8.11–48.43, *p* < 0.001 at baseline and aOR: 8.68, 95% CI 6.13–12.29, *p* < 0.001 at follow-up). Significant occupational risk factors at baseline included exposure to COVID-19 patients (aOR: 2.79, 95% CI 1.28–6.09, *p* = 0.010) and to SARS-CoV-2 infected co-workers (aOR: 2.50, 95% CI 1.52–4.12, *p* < 0.001). At follow up 6 months later, non-occupational exposure to SARS-CoV-2 infected individuals (aOR: 2.54, 95% CI 1.66–3.89 *p* < 0.001) and the local COVID-19 incidence of the corresponding HCI (aOR: 1.98, 95% CI 1.30–3.02, *p* = 0.001) were associated with seroconversion. The healthcare institutions’ mask policy (surgical masks during usual exposure vs. general use of FFP-2 masks) did not affect seroconversion rates of HCW during the first and the second pandemic wave.

**Conclusion:**

Contact with SARS-CoV-2 infected household members was the most important risk factor for seroconversion among HCW. The strongest occupational risk factor was exposure to COVID-19 patients. During this pandemic, with heavy non-occupational exposure to SARS-CoV-2, the mask policy of HCIs did not affect the seroconversion rate of HCWs.

**Supplementary Information:**

The online version contains supplementary material available at 10.1186/s13756-021-01047-x.

## Introduction

Health care workers (HCW) are engaged at the frontline of the COVID-19 pandemic and are thereby heavily exposed to SARS-CoV-2. From the beginning of the pandemic, several studies have investigated the risk for HCW for infections with SARS-CoV-2. While some studies showed low infection rates among HCW indicating that the implemented protection measures were effective [1–4], others studies revealed high infection rates among HCW [5–11]. The most frequently identified risk factors for transmission of SARS-CoV-2 to HCW include working at dedicated COVID-19 units [6, 7], having either direct contact with infected patients [8, 9], or infected co-workers [9, 11] and being exposed to infected household members [8, 10, 11].

There is still an ongoing debate about the mode of transmission of SARS-CoV-2 by respiratory particles. Some advocate that transmission of SARS-CoV-2 mainly occurs by droplets [12, 13] while others highlight the importance of aerosols [14, 15]. Accordingly, there is inconsistency in recommendations regarding the use of different types of masks for protection of HCW. The World Health Organization (WHO), Public Health England, and the Swiss National Centre for Infection Control (Swissnoso) recommended the use of surgical masks, with the exception for exposure during aerosol-generating procedures (AGP) [16–18]. In contrast, the United States Centers for Diseases Control and Prevention (CDC), the European Centre for Disease Prevention and Control (ECDC), and the German Robert Koch Institute recommended universal use of filtering face piece class-2 (FFP-2) masks for protection against airborne transmission [19–21]. Indeed, recent publications suggest that aerosols arise not only during AGP and therefore FFP-2 masks might be advantageous in virus-rich indoor environments including medical centres and hospitals [22–24].

Despite the official recommendation in Switzerland to restrict the use of FFP-2 masks to AGP, about half of health care institutions (HCI) in the Canton of Grisons opted for a general use of FFP-2 masks. We therefore took the opportunity of this particular situation to analyse the effect of the healthcare institutions’ mask policy on SARS-CoV-2 seroconversion rates among HCW.

Thus, the aim of our study was to assess risk factors for SARS-CoV-2 seroconversion among HCW in the canton of Grisons, Switzerland during the first and the second epidemic wave, with a special emphasis on the healthcare institution’s recommendation regarding the use of FFP-2 masks.

## Methods

### Participants and setting

We performed a multicenter prospective cohort study including 13 HCI caring for patients with COVID-19 in the canton of Grisons, Switzerland. The selected HCI consisted of one referral center (provincial hospital; 330 bed medical center with integrated intensive care unit), nine small primary care hospitals (average bed capacity: 33 beds), two rehabilitation centers (average bed capacity: 108 beds) and one psychiatric clinic (capacity of 240 beds). We invited all health care employees (≥ 16 years of age) in the selected HCI to participate in the study.

**Fig. 1:**
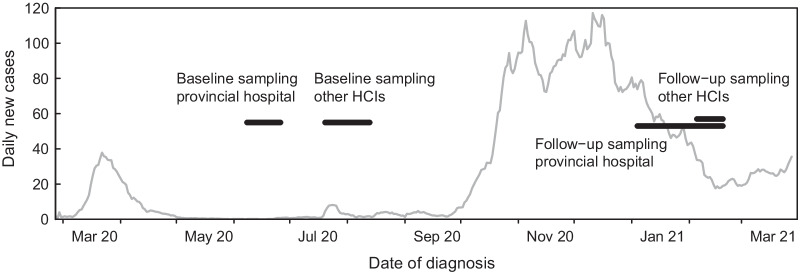
Timeline of the study. The pandemic in the Canton of Grisons from March 2020 to March 2021 is represented by the 7-day running mean of daily numbers of new COVID-19 cases. Horizontal bars indicate the time of baseline and follow up sampling in the participating health care institutions (HCIs)

Study participants underwent serological SARS-CoV-2 testing at baseline and concomitantly completed a questionnaire to assess exposure to anticipated risk factors for SARS-CoV-2 infection after the first epidemic wave (sampling provincial hospital: June 8–June 26, 2020; sampling other HCIs: July 20–August 13, 2020). The study participants underwent the same procedure during the second pandemic wave (follow-up sampling provincial hospital: January 4–February 19, 2021; follow-up sampling other HCIs: February 1–February 19, 2021). The timeline of the study is pictured in Fig. 1. The incidence of COVID-19 per 100,000 inhabitants in the different study regions was retrieved from the health department of the canton Grisons [25]. The study was approved by the ethics committee of the canton Zurich (BASEC-No.: 2020-01322). We obtained written informed consent from all participants before enrollment.

### Serologic testing

We collected serum (10 mL) at baseline and follow-up. SARS-CoV-2 nucleocapsid (N) antibody concentrations were determined using a commercially available electrochemiluminescence immunoassay (ECLIA, Elecsys® Anti-SARS-CoV-2, Roche, Basel, Switzerland), which was run on a COBAS 6000 analyzer (Roche, Rothkreuz, Swizterland). Seropositivity was defined according to the manufacturers’ instructions (cutoff index (COI) > 1) and seroconversion was defined as the first detection of SARS-CoV-2 antibodies above the threshold. The manufacturer-reported sensitivity and specificity (> 14 days after PCR positivity) of the assay is 100% (95% CI 88.1–100%) and 99.80% (95% CI 99.69–99.88), respectively [26].

### Online questionnaire

To assess risk factors for seroconversion, participants were asked to complete an online questionnaire at each time point of serological sampling. We requested participants to fill in the questionnaire at both time points to make sure that changes of risk factors (e.g. occupational exposure to COVID-19 patients) were correctly captured over time. In the baseline questionnaire, study participants were asked to report their possible exposure to SARS-CoV-2 from the beginning of the pandemic until baseline and in the follow-up questionnaire, they were explicit asked to report their possible exposure occurring between baseline and follow up. Variables of interest included sex, age, type of ABO blood group, type of occupation, level of employment, occupational exposure to patients (with or without COVID-19), occupational exposure to SARS-CoV-2 infected co-workers, household and other non-occupational exposure to individuals with COVID-19. Additionally, self-reported results of previously performed SARS-CoV-2 nasopharyngeal swabs were collected (see AMICO Questionnaire in the Additional file 1).

### Preventive measures for healthcare workers

According to the guidelines of the National Centre for Infection Control [16], seven HCI (one secondary care hospital, five primary care hospitals and one rehabilitation clinic) implemented recommendations to use surgical face masks type IIR during usual exposures and restricted the use of FFP-2 masks to AGP. The remaining six other institutions (four primary care hospitals, one rehabilitation clinic and one psychiatric clinic) recommended the general use of FFP-2 masks for all contacts with COVID-19 patients. Other preventive measures for HCW were similar among institutions and included the use of gloves, gowns and goggles during exposure to COVID-19 patients, hand-hygiene measures and social distancing following the Swiss guidelines released by Swissnoso [18] and the Federal Office of Public Health (FOPH) [27]. The compliance of the HCW with the institutional mask policy and the other recommended protective measure was not systematically assessed as part of the study.

No health care institution reported an important lack of personal protective equipment.

### Statistical analysis

Seropositivity rates were reported as proportions. Associations between potential risk factors (personal and professional characteristics, individual COVID-19 exposure, regional incidence, FFP-2 mask policy) and seroconversion were assessed by calculating the proportion of participants with seroconversion for each level of these factors. Odds ratios (OR) and 95% confidence intervals (95% CI) for comparisons between factor levels were determined by logistic regression, and *p* values from Wald tests are reported. For factors determined at institution level (regional incidence, FFP-2 mask policy), regression models included institutions as random effect. The combined effects of individual COVID-19 exposure, regional incidence and FFP-2 mask policy on the probability of seroconversion were evaluated with multivariable logistic mixed-effects models including institutions as random effect. To account for possible confounders, models additionally included factors that showed a significant association with seroconversion at least at one time point, excluding factors that were recorded only for a subgroup of participants. Statistical analysis was performed using the software R, version 4.0.2 (R Foundation for Statistical Computing, Vienna, 2020, www.R-project.org).

## Results

### Study population

**Fig. 2:**
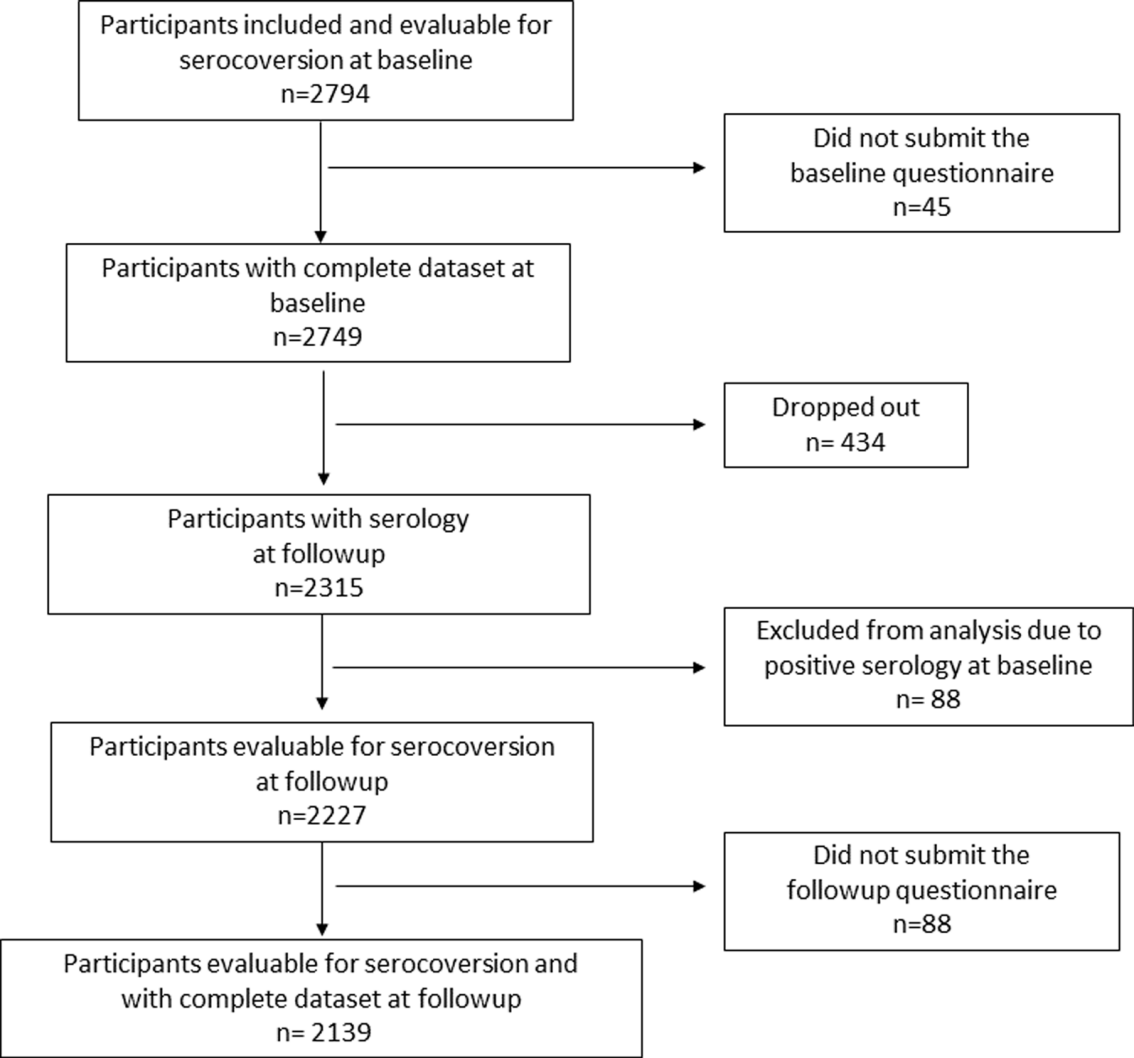
Flow diagram for study participants

We included 2794 HCW from 13 healthcare institutions of the canton Grisons, Switzerland. This corresponds to 49% of all HCW employed in the participating HCI. SARS-CoV-2 serological testing was performed for 100% (2794/2794) of participants at baseline, and for 83% (2315/2794) of participants at follow up (Fig. 2).

Baseline characteristics of study participants according to the HCIs mask policy are summarised in Table 1. The proportion of study participants with occupational exposure to patients with COVID-19 was similar among HCI with different mask policies. The mean regional incidence of COVID-19 at baseline and at follow-up was higher for HCI that recommended general use of FFP-2 masks. An overview of all HCI participating in the AMICO is provided in in the Additional file 1: Table S1.

**Table 1:** Characteristics of study participants according to the mask policy of the respective health care institution

	FFP-2 masks recommended exclusively for aerosol generating procedures in patients with COVID-19	FFP-2 masks recommended for all interactions with patients with COVID-19
Number of health care institutions	7	6
Number of participants (n = 2794)	1993	801
Age, median (IQR)	42 (31–52)	45 (33–54)
Sex: n (%) female	1579 (79.2%)	618 (77.2%)
Profession		
Employee without patient exposure	406 (20.4%)	153 (19.1%)
Nurse	823 (41.3%)	295 (36.8%)
Physician	305 (15.3%)	71 (8.9%)
Other employee with patient exposure	459 (23.0%)	282 (35.2%)
Employee in ICU	168 (8.4%)	19 (2.4%)
Employee in COVID-19 ward	186 (9.3%)	60 (7.5%)
Employee in emergency unit	309 (15.5%)	87 (10.9%)
*Exposure at baseline (n* = *2757)*		
Type of patient exposure		
No patient exposure	601 (30.2%)	244 (30.5%)
Exposure to non-COVID-19 patients	827 (41.5%)	384 (47.9%)
Exposure to COVID-19 patients	532 (26.7%)	169 (21.1%)
Exposure to SARS-CoV2 infected co-worker	297 (14.9%)	86 (10.7%)
Non-occupational exposure to SARS-CoV2 infected person	58 (2.9%)	33 (4.1%)
*Exposure at follow up (n* = *2139)*		
Type of patient exposure		
No patient exposure	499 (30.5%)	210 (35.5%)
Exposure to non-COVID-19 patients	339 (20.7%)	154 (26.1%)
Exposure to COVID-19 patients	729 (44.6%)	208 (35.2%)
Exposure to SARS-CoV2 infected co-worker	322 (19.7%)	123 (20.8%)
Non-occupational exposure to SARS-CoV2 infected person	161 (9.8%)	70 (11.8%)
Cumulative regional incidence per 100,000 inhabitants (July 2020/March 2021), mean	340/5298	539/6432

### Risk factors associated with SARS-CoV-2 seroconversion

SARS-CoV-2 serology was positive in 3.5% (99/2794) of participants at baseline and in 16.2% (376/2315) of participants at follow-up. Eighty-eight participants with positive serology at baseline participated in the follow-up, of whom 97.7% (86/88) remained seropositive. Additional 13% (290/2227) of participants, who were seronegative at baseline, seroconverted until the time of the follow-up.

In univariable analysis, contact to a SARS-CoV-2 infected household member was the strongest risk factor for seroconversion (OR: 20.70, 95% CI 9.43–43.99, *p* < 0.001, at baseline and OR: 9.02, 95% CI 6.43–12.67, *p* < 0.001, at follow-up). Occupational exposure to COVID-19 patients was also associated with seroconversion at both time points. (OR: 3.23, 95% CI 1.89–5.76, *p* < 0.001 at baseline and OR: 1.73, 95% CI 1.29–2.33, *p* < 0.001 at follow-up). Conversely, the healthcare institutions’FFP-2 mask policy was not significantly associated with seroconversion for SARS-CoV-2 (OR: 0.7, 95% CI 0.20–2.40, *p* = 0.567 at baseline and OR: 1.14, 95% CI 0.60–2.16, *p* = 0.693 at follow up). An additional subgroup analysis of HCW with direct exposure to COVID-19 patients did not reveal an association between seroconversion and the healthcare institutions’ mask policy (Additional file 1: Table S2). Occupational risk factors for seroconversion at baseline and at follow-up included: working as a nurse (OR: 2.38, 95% CI 1.28–4.85, *p* = 0.010 at baseline and OR: 1.72, 95% CI 1.23–2.46, *p* = 0.002 at follow-up), working in COVID-19 specific wards (OR: 4.19, 95% CI 2.57–6.69, *p* < 0.001 at baseline and OR: 1.80, 95% CI 1.28–2.51, *p* = 0.001 at follow-up) and exposure to a SARS-CoV-2 positive co-worker (OR: 4.64, 95% CI 3.04–7.04, *p* < 0.001 at baseline and OR: 1.44, 95% CI 1.07–1.92, *p* = 0.015 at follow-up). A high regional incidence of COVID-19 (OR: 2.06, 95% CI 1.10–3.89, *p* = 0.025) and non-occupational contact with SARS-CoV-2 positive individuals (OR: 2.64, 95% CI 1.72–3.95, *p* < 0.001) were only associated with seroconversion at the follow-up measurement (Table 2).

**Table 2:** Risk factors for SARS-CoV-2 seroconversion

	Baseline n = 2794						Follow up n = 2227					
	n total	n sero-conversion	% sero-conversion	OR	95% CI	*p* value	n total	N sero-conversion	% Sero-conversion	OR	95% CI	*p* value
*Age*												
Per 10 years	2794	99		1.10	0.93–1.29	0.267	2227	290		0.99	0.98–1.00	0.104
*Sex*												
Female	2197	76	3.5				1763	227	12.9			
Male	597	23	3.9	1.12	0.68–1.77	0.645	464	63	13.6	1.06	0.78–1.43	0.689
*Blood group*												
0	722	25	3.5				695	74	10.6			
A	693	32	4.6	1.35	0.79–2.32	0.271	654	107	16.4	1.64	1.20–2.26	0.002
B	182	7	3.8	1.12	0.44–2.49	0.802	173	23	13.3	1.29	0.77–2.09	0.324
AB	64	0	0.0	0.00	0.00–15.72	0.977	64	14	21.9	2.35	1.20–4.35	0.009
unknown	1133	35	3.1	0.89	0.53–1.51	0.658	641	72	11.2	1.06	0.75–1.50	0.732
*Employment rate*												
< 60%	514	15	2.9				410	61	14.9			
60–79%	359	14	3.9	1.35	0.64–2.85	0.427	293	38	13.0	0.85	0.55–1.31	0.473
80–100%	1873	69	3.7	1.27	0.74–2.33	0.405	1436	182	12.7	0.83	0.61–1.14	0.245
*Profession*												
Employee without patient exposure	559	11	2.0				478	48	10.0			
Physician	376	9	2.4	1.22	0.49–2.98	0.660	273	28	10.3	1.02	0.62–1.66	0.925
Nurse	1118	51	4.6	2.38	1.28–4.85	0.010	886	143	16.1	1.72	1.23–2.46	0.002
Other employee with patient exposure	741	28	3.8	1.96	0.99–4.14	0.063	590	71	12.0	1.23	0.83–1.81	0.304
*Type of patient exposure*												
None	845	18	2.1				709	74	10.4			
No COVID-19 patients	1211	34	2.8	1.33	0.75–2.41	0.337	493	50	10.1	0.97	0.66–1.41	0.869
With COVID-19 patients	701	46	6.6	3.23	1.89–5.76	< 0.001	937	157	16.8	1.73	1.29–2.33	< 0.001
*Unprotected exposure to COVID-19 patient*												
No	664	42	6.3				880	149	16.9			
Yes	38	4	10.5	1.74	0.50–4.63	0.315	58	9	15.5	0.90	0.40–1.79	0.780
*ICU employee*												
No	1675	67	4.0				1219	177	14.5			
Yes	253	14	5.5	1.41	0.75–2.47	0.259	213	30	14.1	0.97	0.63–1.45	0.868
*Employee at COVID-19 ward*												
No	1681	52	3.1				1151	148	12.9			
Yes	246	29	11.8	4.19	2.57–6.69	< 0.001	281	59	21.0	1.80	1.28–2.51	0.001
*Employee in emergency unit*												
No	1531	57	3.7				1114	166	14.9			
Yes	396	24	6.1	1.67	1.00–2.69	0.041	318	41	12.9	0.85	0.58–1.21	0.37
*Exposure to SARS-CoV2 infected co-worker*												
No	2368	58	2.4				1665	200	12.0			
Yes	383	40	10.4	4.64	3.04–7.04	< 0.001	445	73	16.4	1.44	1.07–1.92	0.015
*Non-occupational exposure to SARS-CoV2 infected person*												
No or unknown	2660	83	3.1				1810	166	9.2			
Household	30	12	40.0	20.70	9.43–43.99	< 0.001	172	82	47.7	9.02	6.43–12.67	< 0.001
Outside household	61	3	4.9	1.61	0.39–4.46	0.432	157	33	21.0	2.64	1.72–3.95	< 0.001
*Child (ren)living same in household*												
No	2081	73	3.5				1635	209	12.8			
Yes	669	25	3.7	1.07	0.66–1.67	0.781	504	72	14.3	1.14	0.85–1.51	0.383
Regional incidence*												
Low	2393	79	3.3				1879	219	11.7			
Medium	141	1	0.7	0.16	0.01–1.94	0.151	129	23	17.8	1.68	0.78–3.61	0.184
High	260	19	7.3	1.88	0.50–7.12	0.353	219	48	21.9	2.06	1.10–3.89	0.025
FFP2 mask policy*												
Specific use	1993	68	3.4				1636	204	12.5			
General use	801	31	3.9	0.70	0.20–2.40	0.567	591	86	14.6	1.14	0.60–2.16	0.693

Participants who seroconverted before the baseline assessment (n = 99) were excluded for analysis of seroconversion at follow-up. Note that total n for individual risk factors may be smaller due to missing values

^*^For these factors determined at institutional level regression models included health care institutions as random effect

**Fig. 3:**
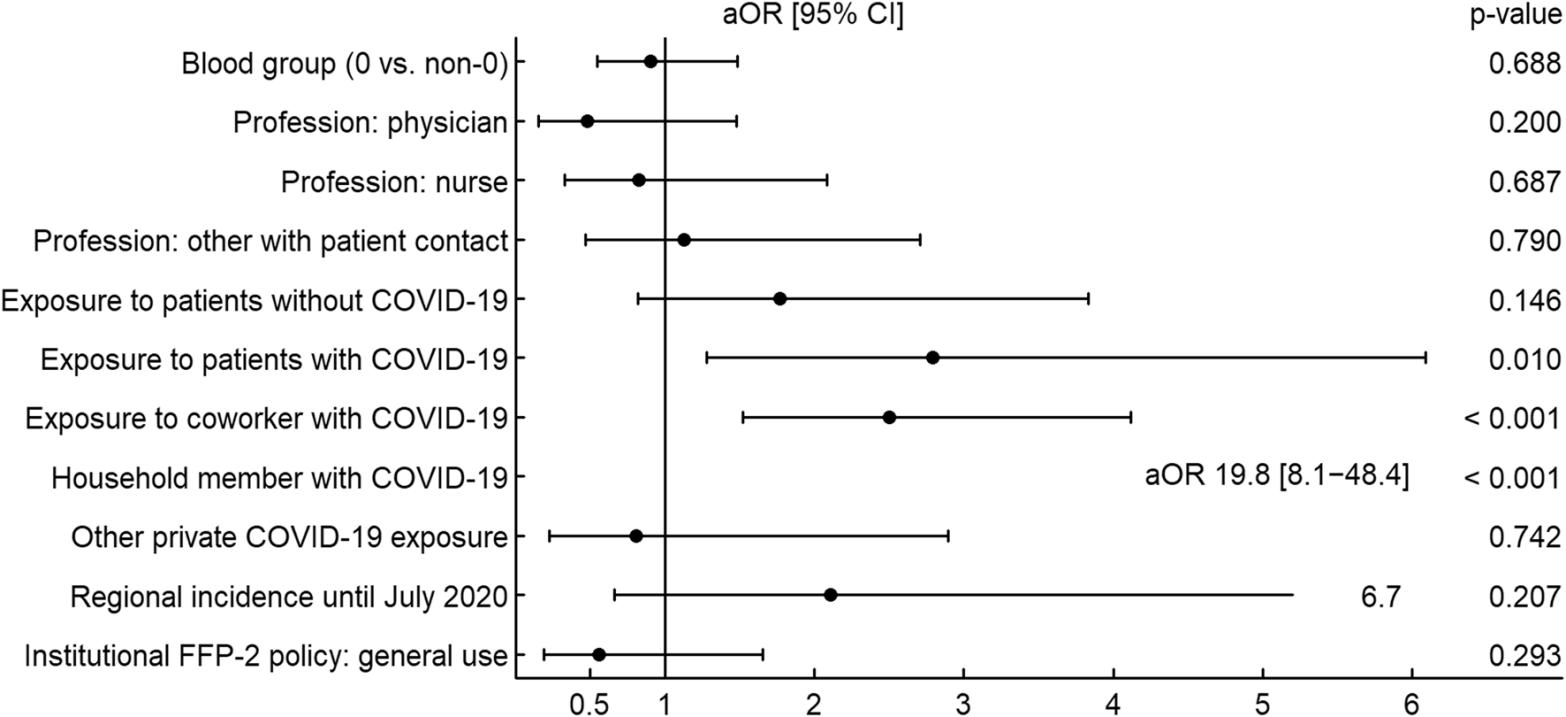
Risk factors associated with SARS-CoV-2 seroconversion (multivariate model) at baseline. Adjusted odds ratios (aOR) and 95% confidence intervals (CI) as well as *p* values (Wald tests) derived from a logistic mixed-effects model for seroconversion at baseline (*n* = 2749)

**Fig. 4:**
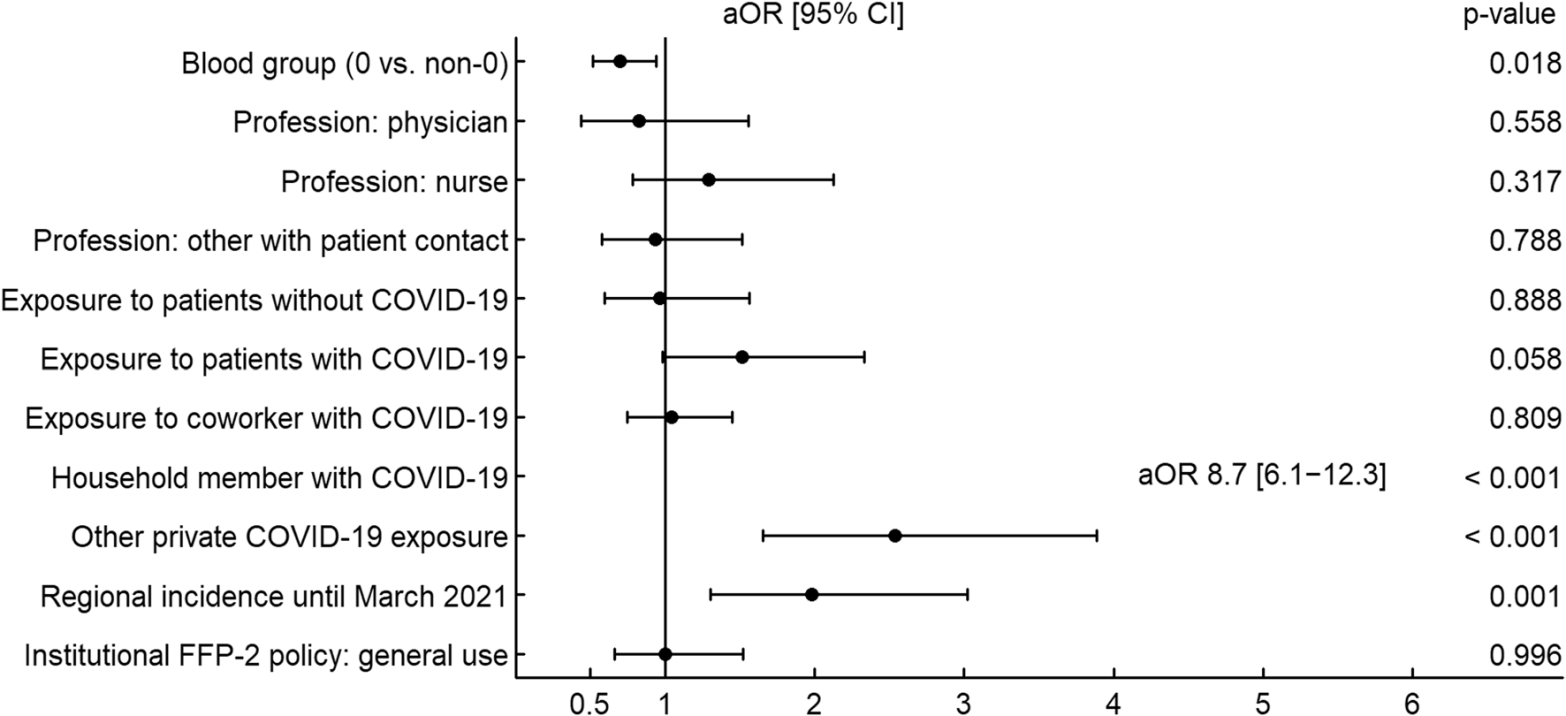
Risk factors associated with SARS-CoV-2 seroconversion (multivariate model) at follow-up. Adjusted odds ratios (aOR) and 95% confidence intervals (CI) as well as *p* values (Wald tests) derived from a logistic mixed-effects model for seroconversion at survey 2 (n = 2139)

In multivariable analyses contact with a SARS-CoV-2 infected household member was still strongly associated with seroconversion (aOR: 19.82, 95% CI 8.11–48.43, *p* < 0.001 at baseline and aOR: 8.68, 95% CI 6.13–12.29, *p* < 0.001 at follow-up). Occupational risk factors at baseline included exposure to COVID-19 patients (aOR: 2.79, 95% CI 1.28–6.09, *p* = 0.010) and contact with a SARS-CoV-2 positive co-worker (aOR: 2.50, 95% CI 1.52–4.12, *p* < 0.001). In contrast, at follow-up, non-occupational contact with SARS-CoV-2 positive individuals (aOR: 2.54, 95% CI 1.66–3.89, *p* < 0.001) and the COVID-19 incidence in the region of the HCI (aOR: 1.98, 95% CI 1.30–3.02, *p* = 0.001) were associated with an increased risk for seroconversion. Interestingly, healthcare workers with the blood group O were less likely to seroconvert (aOR: 0.7, 95% CI 0.52–0.94, *p* = 0.018). Of note, the healthcare institutions’ mask policy was neither associated with seroconversion at baseline nor at the time of follow-up (Figs. 3 and 4). Even in the subgroup of healthcare workers with direct exposure to SARS-CoV-2 infected patients, we did not find an association between seroconversion and the healthcare institutions’ mask policy (Additional file 1: Table S3).

### Association between SARS-CoV-2 PCR test result and seroconversion

Only 17% (479/2749) of participants underwent a nasopharyngeal swab with subsequent SARS-CoV-2 PCR testing before the baseline assessment. At the time of the follow-up 59% (1272/2139) of participants had SARS-CoV-2 PCR testing. Serological testing was positive in 93% (54/58) of participants with positive SARS-CoV-2 PCR at baseline and in 95% (227/239) of participants at follow-up. 45% (45/99) of participants with seroconversion at baseline and 21% (63/290) with seroconversion at follow-up did not report previous positive PCR test results.

## Discussion

In this multicentre prospective cohort study, one sixth of participating HCW were seropositive for SARS-CoV-2 as by early 2021. The most important risk factor for seroconversion was household exposure to a SARS-CoV-2 infected individual. Occupational risk factors such as exposure to COVID-19 patients and contact with SARS-CoV-2 positive co-workers were associated with seroconversion during the first pandemic wave. During the second wave of the pandemic non-occupational contact with persons with SARS-CoV-2 infection and the regional COVID-19 incidence were identified as risk factors for seroconversion. Interestingly, the healthcare institutions’ mask policy (surgical mask vs. FFP-2 mask) did not affect the risk of HCW to seroconvert.

Household exposure to a confirmed COVID-19 case has been reported to be a major risk factor for seroconversion among HCW in previous studies [8, 10, 11]. However, it is difficult to dissect the exact sequence of infections as asymptomatically infected HCW may also transmit the virus to household members who subsequently develop symptomatic disease. A recent Scottish study reported a two-fold increased risk for hospital admissions of household members of HCW compared to the general population [28], suggesting that HCW may play an important role in the spread of SARS-CoV-2.

An interesting finding of our study is the shift from occupation related infections at baseline to non-occupational infections at follow-up. This finding might be related to the adaption of the general preventive measures against COVID-19 in Switzerland after the first pandemic wave: during the first wave, the imposed lockdown reduced social life to a minimum. This might have limited non-occupational transmission significantly. In the later course, loosening the pandemic measures might have led to more non-occupational transmissions among HCW. Accordingly, we found an association between non-occupational contact with persons with SARS-CoV-2 and the regional COVID-19 incidence and seroconversion at follow-up but not at baseline.

A factor that may further have reduced occupational transmissions over time is the increasing number of HCW who reported to have performed a diagnostic PCR test for SARS-CoV-2 from 17% at baseline to 59% at follow up. Accordingly, the number of missed SARS-CoV-2 infections prior to serology decreased from 45 to 21%. Testing of HCW and consequent isolation of infected individuals may have also reduced occupation related transmission over time.

In our study, HCW with direct exposure to COVID-19 patients were at increased risk for seroconversion during the first pandemic wave. This finding is consistent with previous reports [8, 9] and it might reflect important limitations of protective measures probably due to unintentional breaches of safe practice standards or insufficient knowledge in the handling of the personal protective equipment.

Interestingly, we did not find a significant association between the healthcare institutions’ FFP-2 mask policy and the risk for seroconversion among HCW. Even in the subgroup of HCW with direct exposure to COVID-19 patients, we did not find an association. In this point our results differs from those recently published by Haller et al. who reported lower seroconversion rates among HCW with frequent (> 20 patients) COVID-19 exposure, who preferentially used FFP-2 masks [29]. In the study of Haller et al. the authors assessed the use of different mask types individually for each employee, while our study focussed on the mask recommendation on an institutional level.

We observed that HCW with blood group O were less likely to have detectable SARS-CoV-2 antibodies. Several previous studies have also identified an association of the ABO blood group type and the susceptibility to COVID-19 with a protective effect of blood group O [30].

One strength of our study was the comprehensive enrolment of a high proportion of HCW at thirteen HCIs located in the same geographical region. The different FFP-2 mask policies of these institutions, which remained unchanged over time, allowed us to analyse comprehensively their impact on the HCWs’ risk for seroconversion. Additionally, we followed participants longitudinally and performed serological measurements after the first and during the second pandemic wave. This allowed us to analyse the shift in risk factors for seroconversion among HCW during the course of the pandemic.

Some limitations of our study deserve discussion. Infections with SARS-CoV-2 were defined as seroconversion and were methodologically captured with delay. Potentially memory bias in reporting exposure and behaviours may have influenced the results. Moreover, causality between reported exposures and seropositivity can only be assumed but not be proven.

Participating health care institutions were not randomized to different mask policies, but were free to choose their mask strategy. Even though we adjusted our analysis for different confounding factors, the results might have been affected by the non-randomized study design. As HCW were not exclusively exposed to SARS-CoV-2 at work, but also during non-occupational activities, the beneficial effect of FFP-2 masks for occupational safety might have been overlaid by the effect of non-occupational factors. We would therefore like to highlight that our findings regarding the healthcare institutions’ mask policy only apply during periods of heavy non-occupational SARS-CoV-2 exposure of HCW. The higher COVID-19 incidence in the regions where HCI favoured a general use of FFP-2 masks might have additionally diluted the potential protective effect of FFP-2 masks for occupational SARS-COV-2 exposure. As part of the study we have not assed the compliance with mask policy and the other protective measure. Further, the effect of FFP-2 masks in our study may have been limited by scant instructions for safe use of these devices. Especially the lack of systematic mask fitting tests may have affected our results. The inconvenience associated with wearing FFP-2 masks may have also hampered the compliance of employees of the respective healthcare institutions.

## Conclusions

We identified that exposure to SARS-CoV-2 household members was the risk factor with the strongest association for seroconversion among HCW. In addition, HCW with direct exposure to COVID-19 patients were at increased risk for seroconversion. Despite this finding, the healthcare institutions’ mask policy (surgical face masks type IIR vs. FFP-2 masks) had no effect on the proportion of seropositive health care employees at the respective institution during this pandemic with heavy non-occupational SARS-CoV-2 exposure.

## Supplementary Information

**Additional file 1.** Supplementary Data.

## Data Availability

The datasets used and/or analysed during the current study are available from the corresponding author on reasonable request.

## Abbreviations

AGP: Aerosol-generating procedures
CDC: Centers for diseases control and prevention
CI: Confidence interval
COI: Cutoff index (COI)
COVID-19: Corona virus disease-2019
ECDC: European centre for disease prevention and control
ECLIA: Electro-chemiluminescence immunoassay
FFP-2: Filtering face piece class-2
FOPH: Federal office of public health (FOPH)
HCI: Health care institutions
HCW: Health care workers
IgG: Immunoglobulin G
mL: Milliliter
OR: Odds ratio
aOR: Adjusted odds ratio
*p*: *P*-value
PCR: Polymerase chain reaction
PPE: Personal protective equipment (PPE)
SARS-CoV-2: Severe acute respiratory syndrome coronavirus 2
Swissnoso: Swiss national center for infection control
WHO: World Health Organization (WHO
