# Supplementary material for: Healthcare institutions’ recommendation regarding the use of FFP-2 masks and SARS-CoV-2 seropositivity among healthcare workers: a multicenter longitudinal cohort study
Source: Antimicrob Resist Infect Control. 2022 Jan 10;11:6. doi: 10.1186/s13756-021-01047-x (PMC8744038; doi:10.1186/s13756-021-01047-x)
Supplement: Supplementary file 1 — Additional file 1. Supplementary Data. [file 13756_2021_1047_MOESM1_ESM.docx]

**Additional file 1: Data**

**1. Participating Health Care Institutions**

|  | **HCI 1** | **HCI 2** | **HCI 3** | **HCI 4** | **HCI 5** | **HCI 6** | **HCI 7** | **HCI 8** | **HCI 9** | **HCI 10** | **HCI 11** | **HCI 12** | **HCI 13** |
| --- | --- | --- | --- | --- | --- | --- | --- | --- | --- | --- | --- | --- | --- |
| Type of Health Care Institution (HCI) | SCH | PCH | PCH | PCH | PCH | PCH | PCH | PCH | PCH | PCH | RC | RC | PC |
| Number of hospital beds | 330 | 35 | 35 | 71 | 8 | 41 | 45 | 3.5 | 13 | 38 | 156 | 60 | 239 |
| Cumulative Incidence of COVID-19 per  100 000 inhabitants per  July 31 2020 | 317 | 1287 | 245 | 265 | 123 | 245 | 391 | 391 | 1447 | 1287 | 245 | 245 | 317 |
| Cumulative Incidence of COVID-19 per  100 000 inhabitants per  March 15 2021 | 5183 | 9382 | 4792 | 3151 | 4532 | 4792 | 6207 | 6207 | 12530 | 9382 | 4792 | 4792 | 5183 |
| Number of study participants at baseline | 1452 | 123 | 151 | 119 | 49 | 99 | 85 | 56 | 74 | 63 | 174 | 49 | 300 |
| Number of study participants at follow up | 1218 | 117 | 131 | 108 | 47 | 77 | 81 | 49 | 71 | 49 | 96 | 44 | 227 |
| Number (%) of seropositive participants at baseline | 29 (2%) | 12 (10%) | 2 (1%) | 26 (22%) | 0 (0%) | 2 (2%) | 0 (0%) | 1 (2%) | 5 (7%) | 2 (3%) | 3 (2%) | 4 (8%) | 13 (4%) |
| Number (%) of seropositive participants at follow up | 160 (11%) | 27 (22%) | 14 (9%) | 31 (26%) | 4 (8%) | 17 (17%) | 8 (9%) | 16 (29%) | 30 (41%) | 9 (14%) | 20 (11%) | 8 (16%) | 32 (11%) |
| Use of FFP-2-masks | only APG | general use | only APG | only APG | only APG | only APG | general use | general use | only APG | general use | general use | only APG | general use |

**Additional file 1: Table S1: Overview of all Health Care Institution participating in the AMICO study.**

Abbreviations: HCI= Health Care Institution, SCH= Secondary Care Hospital, PCH= Primary Care Hospital, RC= rehabilitation clinic,

PC= psychiatric clinic

**2. Statistical Analysis exclusively for participants with exposure to COVID-19 patients**

**Additional file 1: Table S2: Univariable Analysis of FFP-2 mask policy for SARS-CoV-2 seroconversion exclusively for participants with exposure to COVID-19 patients**

|  |  | Baseline | | | | | | | Follow-up | | | | | | |
| --- | --- | --- | --- | --- | --- | --- | --- | --- | --- | --- | --- | --- | --- | --- | --- |
|  |  | **n total** | **n sero-+** | **% sero-+** | **OR** | **95% CI** | **p-value** | **n total** | | **n sero-+** | **% sero-+** | | **OR** | **95% CI** | **p-value** |
| FFP2 mask policy* | specific use | 532 | 30 | 5.6% |  | | | 729 | | 112 | 15.4% |  | | | |
|  | general use | 169 | 16 | 9.5% | 1.20 | 0.21−6.96 | 0.842 | 208 | | 45 | 21.6% | | 1.26 | 0.56−2.85 | 0.576 |

**Additional file 1: Table S3: Multivariable Analysis of risk factors for SARS-CoV-2 seroconversion exclusively for participants with exposure to COVID-19 patients**

|  | Baseline | | |  | | Follow-up | | | |  |
| --- | --- | --- | --- | --- | --- | --- | --- | --- | --- | --- |
|  | aOR | 95% CI | p-value | |  | | aOR | 95% CI | p-value | |
| Blood group (0 vs. non-0) | 1.08 | 0.52 - 2.26 | 0.832 | |  | | 0.71 | 0.47 - 1.06 | 0.096 | |
| Profession: physician* | 0.20 | 0.01 - 2.83 | 0.237 | |  | | 1.17 | 0.30 - 4.56 | 0.820 | |
| Profession: nurse* | 0.49 | 0.04 - 5.55 | 0.565 | |  | | 1.62 | 0.44 - 5.95 | 0.468 | |
| Profession: other with patient contact* | 0.49 | 0.04 - 6.02 | 0.573 | |  | | 1.11 | 0.29 - 4.23 | 0.878 | |
| Exposure to coworker with COVID-19 | 2.26 | 1.11 - 4.57 | 0.024 | |  | | 0.83 | 0.53 - 1.28 | 0.388 | |
| Household member with COVID-19 | 8.28 | 2.13 - 32.09 | 0.002 | |  | | 4.78 | 2.92 - 7.84 | 0.000 | |
| Other private COVID-19 exposure | 0.75 | 0.14 - 4.18 | 0.745 | |  | | 1.80 | 0.97 - 3.34 | 0.064 | |
| Regional incidence until July 2020 | 1.07 | 0.19 - 6.10 | 0.943 | |  | | 2.20 | 1.06 - 4.57 | 0.034 | |
| Institutional FFP-2 policy: general use | 1.30 | 0.27 - 6.33 | 0.746 | |  | | 1.10 | 0.52 - 2.33 | 0.795 | |

* Reference: profession without patient contact

*Both analyses were done with mixed-effects models including sites as random effect.
